# Key Improvements in Healthcare for Persons With Diabetes at Risk of Developing Foot Ulcers: A Concept Mapping Study

**DOI:** 10.1002/jfa2.70205

**Published:** 2026-09-03

**Authors:** Ulla Hellstrand Tang, Leif Sundberg, Susanne Andersson, Carina Folkesson, Lisbeth Hagström, Magdalena Annersten Gershater

**Affiliations:** ^1^ The Department of Prosthetics & Orthotics Department for Biomedical Engineering and Medical Physics Sahlgrenska University Hospital Gothenburg Sweden; ^2^ Department of Orthopaedics Institute of Clinical Sciences, at the Sahlgrenska Academy at University of Gothenburg Gothenburg Sweden; ^3^ Gothenburg Diabetes Association Gothenburg Sweden; ^4^ Department of Health Sciences University West Trollhättan Sweden; ^5^ R&D Centre Skaraborg, Primary Health Care Skövde Sweden; ^6^ Department of Care Science Faculty of Health and Society Malmö University Malmö Sweden

**Keywords:** concept mapping, diabetic foot, healthcare, podiatry, prevention, self‐care

## Abstract

**Background:**

Diabetic foot ulcers (DFUs) are a serious complication of diabetes, with a lifetime risk of up to 34%. Despite preventive guidelines, gaps remain in the care chain. This study aimed to identify and prioritise improvements in DFU prevention, care, and self‐care in the Region Västra Götaland, Sweden.

**Methods:**

A participatory action research design was used as part of a larger observational co‐design study. Patients with diabetes, healthcare professionals, and researchers participated. Data were collected through Concept Mapping to generate and cluster ideas, followed by a workshop to prioritise improvements. The improvements were assessed regarding feasibility and importance ranging from 0 to 7, with exceptions that some participants scored ideas > 7.

**Results:**

Thirty‐one participants contributed and based on 83 ideas five clusters were generated: (1) Examination of the feet by healthcare professionals, (2) Exchange of knowledge/Exchange of experiences, (3) Access to podiatry, (4) Economy/Equal care, and (5) Other/Support for self‐care. In a workshop with 22 participants, the three prioritised ideas were: (1) *Having access to podiatry if problems occur*, (2) *Getting help when problems arise,* and (3) *Information from the healthcare about the importance of good foot healthcare*. These ideas got 80% or more of all votes within each of the five clusters and were scored > 5 for feasibility and of > 7 for importance. Other prioritised ideas involved enhancing individual self‐care capabilities and maintaining personalised interactions despite digitalisation.

**Conclusions:**

Timely access to podiatry, increased foot health awareness among healthcare professionals, and rapid intervention when problems arise were identified as key priorities for improving DFU care and patient outcomes.

**Abbreviations**
DFU: diabetic foot ulcer
DPO: Department of Prosthetics and Orthotics
HCA: hierarchical cluster analysis
HCP: healthcare professional
IWGDF: International Working Group on the Diabetic Foot
PI: Principal Investigator
VGR: the Region Västra Götaland

## 1 Introduction

Worldwide, 540 million people have diabetes [1], and 0.5 million of them live in Sweden [2]. More than 50% are at high risk of developing diabetic foot ulcers (DFUs) due to neuropathy [3]. Every 20 seconds, an amputation occurs worldwide, caused by DFUs [4, 5]. The lifetime risk of getting DFUs is estimated to be as high as 19%–34% [6], and with comorbidities, the DFU risk is increased [7]. A significant reduction of amputations can be achieved by implementing multi‐disciplinary foot clinics [8], leading to improved quality of life for the patients and reduced healthcare costs [6, 9, 10].

**FIGURE 1:**
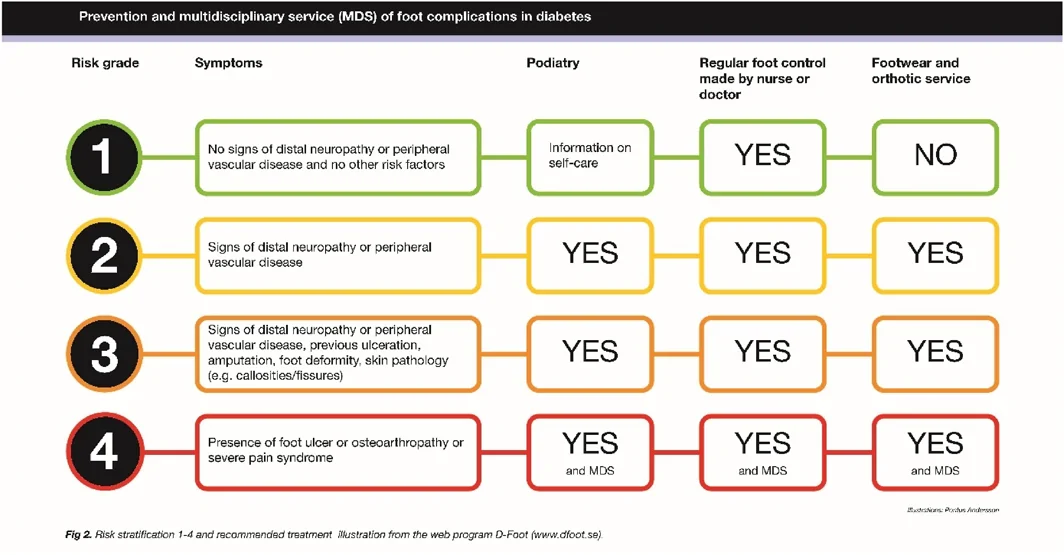
A schema of the risk grades, the symptoms, and the recommended interventions to prevent and treat diabetic foot ulcers. The risk schema [13] is based on national clinical guidelines [11].

In the prevention, as recommended by the International Working Group on the Diabetic Foot (IWGDF) [10] and Swedish guidelines [11, 12], patients should get an annual structured foot examination including a grading of the risk of developing DFU, risk grade 1–4 (1 = no risk of developing DFU to 4 = ongoing DFU) (Figure 1). Based on the risk grading, a care plan is negotiated between the patient and the healthcare professional (HCP) [12]. The care plan should include a description of what actions of self‐care the patient is responsible for and what actions the HCP are responsible for. In addition, to motivate the patient by increasing their knowledge and insights about the need for self‐care, patients at high risk (risk grade 2–3) should be referred to podiatry and to a Department of Prosthetics and Orthotics (DPO) to be counselled regarding appropriate footwear to wear, both indoors and outdoors. Patients with an ongoing DFU should be referred to a multidisciplinary foot clinic, where the team provide medical treatments, local wound care, customised advice of self‐care, podiatry, and offloading devices. Although an annual foot examination is recommended, 25% of all patients with diabetes in Sweden did not receive a foot examination, according to the Swedish National Diabetes Register in 2025 [14].

Comorbidities that come with aging, such as visual impairment and limited mobility, often necessitate assistance with self‐care for people with diabetes [15, 16]. Person‐centred, coordinated care is essential, as highlighted in recent clinical guidelines for those at high risk of foot ulcers [12]. The fact that patients living in assisted living facilities are often left behind when it comes to regular checks, is a challenge, as is reported that in 2020, 82% of all municipality home care in Sweden did not routinely examine older people's feet with the purpose to detect patients at risk and to prevent DFUs [17].

The global transition to a more person‐centred care [18], embracing all HCPs in the care chain, is found to have advantages regarding health economics and quality of life, as reported by Pirhonen et al. in 2020, studying patients with chronic obstructive pulmonary disease and/or chronic heart failure [19]. Little is known of what ideas and what top priorities the patients and the HCPs carry in their mind regarding the transitionto a person‐centred care for patients at risk of developing DFUs. The aim of the study was to map ideas for improvements regarding prevention, management, and self‐care to prevent DFUs, as well as prioritising these ideas.

## 2 Method

### 2.1 Design and Setting

In a participatory action study design, patients living with diabetes, HCPs, and researchers were included. The study was part of a larger observational co‐design study—‘Optimised care of people with diabetes and foot complications in primary care’—that was carried out in Region Västra Götaland (VGR) [20]. The main part of this study was conducted in Skaraborg county, located in the southwest of Sweden.

### 2.2 Participants

The inclusion criteria for the participating patients were age ≥ 18 years, diagnosed with diabetes, and understanding the Swedish language. Patients were recruited as a convenience sample from three populations, with the aim of collecting ideas for improvement: (1) those who attended a public meeting in Gothenburg on the World Diabetes Day in 2021 [21], (2) those who attended a digital/physical workshop in the VGR in December 2021, and (3) a cohort of patients living with diabetes at five locations in Skaraborg county, in the VGR. Out of 82 eligible participants, 31 people with diabetes accepted to participate in the initial phase, to generate ideas for improvements in the care chain. The inclusion criterion for participation for HCPs was to work with people with diabetes in healthcare. Out of 16 eligible participants, four HCPs generated ideas in the initial phase.

### 2.3 The Iterative Process

**FIGURE 2:**
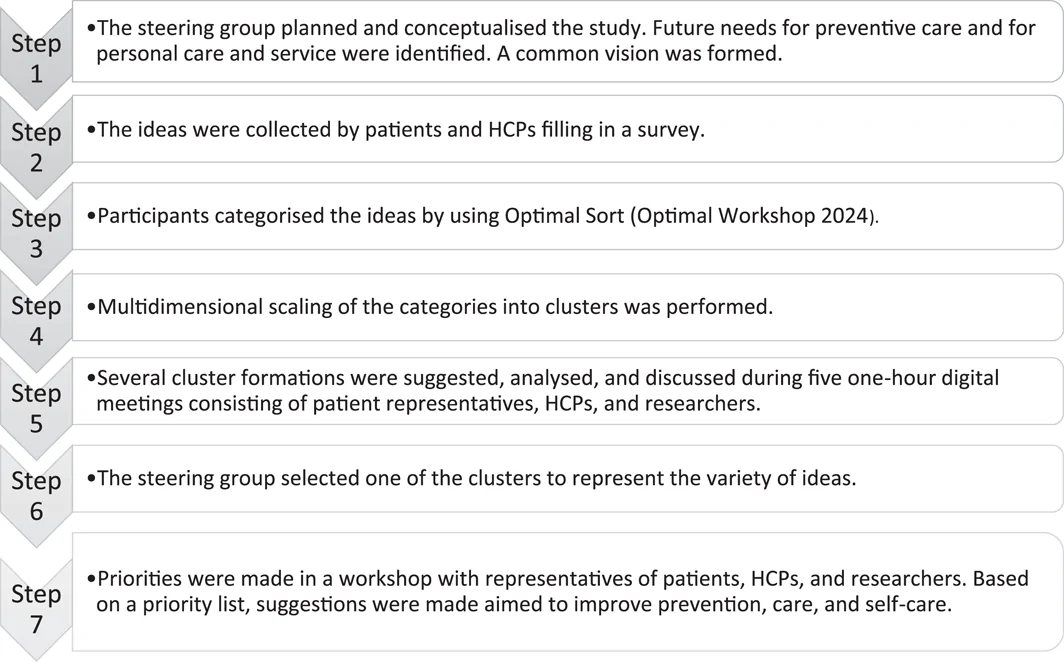
Study flow presenting the steps in the study.

At study start, the steering group planned and conceptualised the study and the rationale, to get insights regarding prioritised improvements in the prevention of DFUs. Thereafter, patients and HCPs answered a survey using Concept Mapping, an action‐oriented research method where those affected gather ideas and create categories [22, 23, 24]. In an iterative process that included collecting ideas for improvement, clustering ideas, and then prioritising ideas, suggestions aimed to improve the prevention of DFUs were made (Figure 2).

In the second step, the study participants were asked to complete an incomplete sentence (a so‐called prompt). The prompt was: ‘To improve podiatry and self‐care in diabetes, I think that…’ (Appendix 1). The participants were asked to fill in a survey in paper format, by means of which their ideas were collected. After the initial data collection, the principal investigator (PI) conducted a screening process to arrive at a final set of ideas that represented the breadth of the incoming suggestions. The screening involved:

- splitting idea,s that contained two or more ideas, into unique ideas.
- removing ideas that were clearly out of scope.
- removing identical or similar ideas while ensuring that no unique idea was excluded.
- reviewing spelling and correcting grammar, if needed.

Through this process, a final set of 83 clearly formulated unique ideas was reached, prepared for the sorting stage.

In step 3, a group of participants, consisting of one patient living with diabetes and four HCPs, sorted and categorised the ideas individually by using the software tool Optimal Sort [25]. The participants were asked to categorise the unique ideas into themes, based on their own perception of similarities and content. The sorting was considered complete when each participant had sorted the ideas into a category.

In the fourth step, the Multidimensional Scaling (MDS) algorithm [26] was applied to approximate the sorted ideas as distances and assign coordinates to the ideas in two dimensions. By using this algorithm, a map was generated, where the distance between ideas that often seem to belong together was short, and the distance between ideas that seldom seem to belong together was long. In the MDS, a so‐called stress value provides an indication of how well the points in two dimensions represent the sorted data. A stress value below a given threshold for the same number of points in the same dimensions, suggests that the likelihood of the sorting being arbitrary is lower than 1%. Clusters were identified on the map using hierarchical cluster analysis (HCA). The goal was to facilitate interpretation of the map while representing the content of the ideas in a meaningful way.

In the fifth step, the steering group reviewed the cluster solutions sequentially, and in the sixth step, the project team decided which cluster solution was appropriate to proceed with in the next, the prioritising phase. In a seventh step, patients with diabetes (*n* = 13), HCPs (*n* = 6), two relatives, and one participant defined as ‘other’, that is, having another profession, participated in an additional workshop, at Skövde Hospital, to further prioritise the ideas for each of the clusters. Out of the six HCPs, five persons worked in municipal care and one person was a podiatrist. Mean age was 67 ± 14; the youngest participant was 30 years old and the oldest participant 81 years old.

### 2.4 Clusters

**FIGURE 3:**
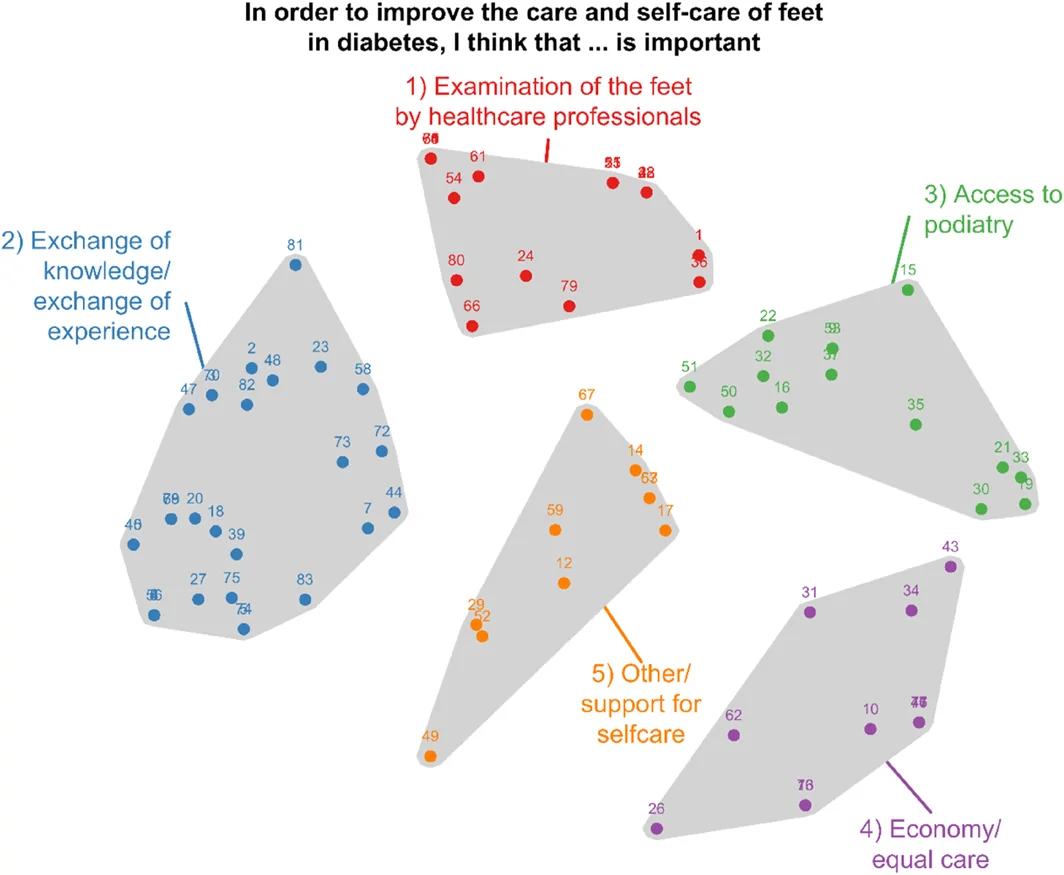
Five clusters representingthe ideas gathered by healthcare professionals and patient representatives. The calculated stress value of the hierarchical cluster analysis was 0.191 for the 83 ideas. Each dot has a number that represents one of the 83 ideas. Examples of the ideas are presented in Appendix 2.

A cluster map consisting of five clusters based on the 83 ideas, was generated (Figure 3). The calculated stress value was 0.191 for the 83 ideas. The titles of the clusters were:

1. Examination of the feet by healthcare professionals
2. Exchange of knowledge/Exchange of experiences
3. Access to podiatry
4. Economy/Equal care
5. Other/Support for self‐care

### 2.5 Workshop to Prioritise the Ideas

In the workshop at Skövde Hospital, participants were organised in five mixed groups with patients with diabetes and HCPs. A group leader, one of the persons in the steering group, was assigned for each group with the task to note down the prioritisation being made in each group. The cluster map, consisting of the five clusters that were considered to visualise the 83 ideas, was presented to the participants. The participants worked for three hours to prioritise among the 83 ideas. First, they individually prioritised the ideas. Thereafter, based on a group discussion, each group prioritised the ideas in each cluster, with between 10 and 29 ideas per cluster. The participants had an option to add ideas to each of the clusters and to comment on the priorities that were made. They work started with cluster 1, entitled ‘Examination of the feet by healthcare professionals’, and participants selected the most important ideas and registered those ideas in a paper template. Subsequently, the participants were instructed to score the chosen ideas according to feasibility and importance on a scale from one to seven (1 = completely unimportant to 7 = very important) (Table 1). In addition to written recording with pen and paper, the workshop was audio recorded so that it would be possible to recall the discussion. All participants signed a written informed consent prior to the start of the workshop.

**TABLE 1:** Ideas, from the group work, that got most votes (80% or more), out the total number of ideas in each cluster.

1. Idea in English (and Swedish)	2. Cluster	3. Per cent of votes out of the total number of ideas in each cluster (%)	4. Feasibility	5. Importance
One should have access to podiatry if problems occur. (Man ska få fotvård vid upplevt problem.)	3	100	6.0	7.4
Getting help if I have problems (Få hjälp om jag får problem)	1	80	5.0	7.3
The healthcare should inform about the importance of good healthcare (Vården bör informera om vikten av god fothälsa.)	2	80	7.0	7.0
Regular check‐ups by healthcare/healthcare provider (Regelbundet bli kallad till vården)	3	80	6.3	6.5
Gettingtimely care (Att man får vård i rimlig tid)	3	80	5.7	7.5
Solutions for how home care from the municipality is organised regarding assisted self‐care of the feet should be as natural as having assisted care for showering and cutting one's finger nails. (Man behöver se hur man löser individens möjlighet att klara egenvården rent fysiskt om man inte har SOL [socialtjänstlagen/UT]‐insatser eller bor på Säbo)	4	80	6.3	6.8
Orthotics, e.g., for between toes, should be recommended (Bör rekommendera lämpliga inlägg, t.ex. mellan tårna.)	5	80	6.8	6.8
Getting help with insoles by healthcare providers working at a Department of Prosthetics & Orthotics. (Få hjälp med utprovning av inlägg av ortopedteknisk personal)	5	80	6.7	7.0
Ensuring that the personal meeting is maintained, even if digital consulting is implemented. (Att det personliga mötet inte får försvinna, tunnas ut, när digitala stöd införs)	5	80	6.3	5.8
We need to see and understand the view of the patient. (Vi behöver se/förstå var patienten befinner sig)	5	80	7.0	7.0

*Note:* The first column presents the idea. The second column presents the number of the cluster, from 1 to 5 (see Figure 3). The third column shows, in percentage, the votes divided by the total number of clusters (*n* = 5). The fourth column presents the feasibility, and the fifth column the importance, of the idea (0‐7, however some participant scored > 7).

## 3 Results

The three prioritised ideas were: (1) Having access to podiatry if problems occur, (2) Getting help when problems arise, and (3) Information from the healthcare about the importance of good foot healthcare. These ideas got 80% or more of all votes within each of the five clusters and were scored > 5 for feasibility and of > 7 for importance. Other prioritised ideas involved enhancing individual self‐care capabilities and maintaining personalised interactions despite digitalisation. The prioritisation for each cluster is presented below.

In cluster 1, with the theme ‘Examination of Feet in Healthcare’, the groups scored the quote ‘*Getting help if problems arise’* as important. Introducing regular foot examinations early after diagnosis, and the importance for healthcare providers to have good knowledge about the diabetic foot, were also emphasised. The groups commented that accessibility of healthcare and standardisation of foot examinations should be considered.

In cluster 2, with the theme ‘Exchange of knowledge/Exchange of experiences’, the group scored *Information about the diabetic foot and the importance of good foot healthcare for people with diabetes as* the most important thing for healthcare providers. Advice should be based on the patient's actual foot status. It was also mentioned as important to motivate patients to take responsibility for their own foot health. The groups commented that it was important for patients to learn how to perform self‐care, preferably through diabetes education programmes.

In cluster 3, with the theme ‘Access to Podiatry’, the need to *receive podiatry when experiencing problems* was prioritised. Participants also considered that it was important for patients to be regularly followed up and to receive podiatry within a reasonable time frame. From the groups' comments: More resources are needed so that patients at risk of DFU receive the necessary care, such as help cutting their toenails. Patients should not be referred to a private podiatrist for the cutting of their nails. This service should be provided by the municipality.

Cluster 4, with the theme ‘Economy/Equal Care’, addressed the fact that solutions were needed to organise home care from the municipality regarding assisted self‐care of the feet. This should be included as routine help, similar to when persons need help with personal hygiene, such as assisted care for showers and nail care of the hands. In the workshop, the group highly prioritised the idea that primary healthcare centres should refer to podiatry and that a podiatrist should be included in the diabetes teams. In addition, podiatry should be subsidised. Access to better shoes and insoles was also on the wish list. The groups commented that it is important to educate the municipality social workers regarding the special needs that patients with diabetes might have, in order to enable a fair evaluation regarding assistance.

In Cluster 5, with the theme ‘Other/Support for Self‐Care’, the participants emphasised that it is important to understand the patient's situation (mentally and physically). At a care visit, it is important to have a personal meeting with HCPs. Personal meetings should not disappear, or be spaced out, when digital support is introduced. In addition, receiving help and recommendations in a language that the patients understand, was seen as important, as was the fitting of insoles and other orthotics by certified prosthetists and orthotists. Moreover, HCPs working in home care or in nursing homes should assist persons with self‐care of the feet. The groups commented that a clear division of responsibilities in healthcare is needed to ensure that tasks are performed as prescribed.

Regarding the individual prioritisation, only one idea was above the 80% threshold, and that ideaand that idea was *Getting help when problems arise*, which got 86% of the votes for the ideas in the same cluster, with a corresponding feasibility of 6.5 and an importance of 7.1. All other ideas were ranked below 80% regarding importance.

## 4 Discussion

The aim of this study was to map and prioritise ideas for improving the prevention, management, and self‐care of the feet of patients with diabetes. The findings emphasise the need for timely and accessible podiatry care, reinforcing the importance of structured healthcare interventions and policy improvements. The quote ‘*Getting help when I need it, having access to podiatry when I have a problem and getting timely help’* summarises the top three ideas with the original themes: (1) *Having access to podiatry if problems occur*, (2) *Getting help when problems arise,* and (3) *Information from the healthcare about the importance of good foot healthcare*. The quote is based on the ideas that got most votes and that were most highly ranked with regard to importance and feasibility (Table 1). In addition, the group prioritised the idea that healthcare overall should emphasise the importance of good foot health and ensure that individuals receive podiatry when they experience problems. The participants also found it essential that people are regularly reminded of their healthcare appointments, and that individuals are provided with insoles from the DPO. Furthermore, while digital support is becoming more prevalent, personal interaction should not be diminished or replaced. Additionally, it is, the participants stated, crucial to understand where the patient is, in their own healthcare journey, in order to provide the most effective support.

*To get help when I need it, to have access to podiatry when I have a problem,* and *to get timely help*—the quote mirror the need and vulnerable position that people with diabetes have to deal with on a daily basis. The phenomenon of vulnerability, which needs to be considered, was revealed in a qualitative study of people with diabetes living with healed foot ulcers [27]. Challenging situations are also described in national guidelines for patients at risk of developing foot ulcers [12]. In those guidelines, it is described how patients with additional illnesses, such as those needing dialysis or eye treatments, find it particularly challenging to balance diabetes‐related foot care with other medical needs. Many patients struggle with mobility issues, making it hard to reach their feet for self‐care. Impaired vision and obesity further complicate foot maintenance and the frequent medical visits disrupt work and social life. Although the national guidelines recommend prevention and care aimed to solve these challenges [11, 12], the 21 independent regions in Sweden encounter problems when it comes to implementation [28]. Only four regions report that they to a high extent offer a care that is in accordance with the national guidelines [28]. Eight regions report that they to a quite high extent, or to some extent, work according to the guidelines. In the same report, the regions declare that working methods, cooperation, and routines, as well as competence and personnel, need to be optimised.

We suggest that the prioritised ideas in the current study form a base for future improvements with regard to implementing the national guidelines in each of the 21 regions. According to Jeffcoate et al., countries around the world face similar problems when trying to implement the IWGDF guidelines [29]. In their study, they found that nationally and internationally the challenges are to form a person‐centred care, create a seamless care flow between organisations, and increase competence [29].

Patients with diabetes presenting with vulnerable feet, in municipal care, often fall between the cracks. In Sweden, assisted self‐care, including personal hygiene for people in need, is provided according to the standards in the Social Services Act [30], which has other goals than the Health and Medical Services Act [31]. An individual who lives at home alone and is unable to manage personal care and has home care services, including shower assistance, might, despite this, not receive assisted self‐care of their feet. Lack of assisted self‐care of the feet might be the case in other countries as well, where the burden of providing assisted self‐care lies on family members, often without any education in diabetes [32].

### 4.1 Discussion of Method and Methodological Considerations

The method used for generating the ideas, Concept Mapping, was found useful when systematically integrating group processes, like brainstorming and idea sorting, with advanced statistical methods, to produce clear, quantitative results. Several steps were taken, for example, brainstorming, sorting of ideas, multidimensional scaling, and HCA [22] before the prioritisation of the ideas was managed in the workshop. The calculated stress value of the HCA was 0.191, which represents the ideas as distances with assigned coordinates in two dimensions [26]. With a stress value of 0.191, the sorting process was considered structured and not random, as the corresponding thresholds in two dimensions were 0.390 for 83 points. According to Trochim and McLinden [33], a larger cluster represents a broader meaning, and clusters located higher up on the map have a higher rating than clusters located lower. Three of the clusters, (1) Examination of the feet by healthcare professionals, (2) Exchange of knowledge/Exchange of experiences, and (3) Access to podiatry – were interpreted, from the cluster map in Figure 3, as being of importance and having a broad meaning.

Comments from the participants regarding the workshop was that they valued the exchange of experiences and getting diverse perspectives from both patients and HCPs. The workshop helped broaden their understanding of foot‐related issues, especially in diabetic care, emphasising the importance of podiatry and health. Moreover, there is a need for greater awareness and prioritisation of podiatry in healthcare, as it is often overlooked and underfunded. During the workshop, differences in healthcare quality were noted across different regions. Suggestions for improvements were to have a larger conference room for future workshops and to get more information about the project prior to the workshop. The participants emphasised that the good ideas should be preserved and built on when optimising prevention, care, and self‐care for patients at risk of developing DFUs.

### 4.2 Participants

Participants included a wide range of stakeholders, from patients and HCPs to researchers, all 18 years or older. Eligible patients and HCPs came from the western part of Sweden, the VGR, representing inhabitants living in both rural areas and cities. A strength is that the participants in the workshop represented a great variety of HCPs from municipal care, primary care, and specialist care. A limitation of the study however, is that age was not registered for all participants.

### 4.3 Context

The findings from this study are relevant in the healthcare context for enhancing the prevention and management of DFUs. The emphasis on receiving prompt and accessible podiatry when experiencing problems, aligns with the necessity to address foot complications early, thereby reducing the risk of severe outcomes like ulcers or amputations. Highlighting the need to educate patients and caregivers underscores the importance of empowering individuals to take responsibility for their foot health, leading to better outcomes. The recognition that digital support should complement—not replace—personal interactions, respects the value of human connection in care delivery.

## 5 Conclusion

Ensuring that individuals with diabetes receive podiatry promptly when they experience problems is critical. The importance of timely and accessible podiatry was the most highly prioritised idea, emphasising the need for accessible healthcare services that address issues early to prevent complications like ulcers. Enhanced patient education and support for self‐care by regular education about foot health and self‐care are essential, considering the patients' individual needs and understanding. Healthcare providers should motivate and support patients to actively manage their foot health, while providing clear, actionable advice. The participants emphasised that the good ideas they suggested should be preserved and built on when optimising prevention, care, and self‐care för patients at risk of developing DFUs.

## Author Contributions

**Ulla Hellstrand Tang:** conceptualization, methodology, software, validation, formal analysis, investigation, resources, data curation, writing – original draft, writing – review and editing. **Leif Sundberg:** conceptualization, methodology, validation, formal analysis, data curation, writing – original draft, writing – review and editing. **Susanne Andersson:** methodology, formal analysis, writing – original draft, writing – review and editing. **Carina Folkesson:** methodology, formal analysis, writing – original draft, writing – review and editing. **Lisbeth Hagström:** methodology, formal analysis, data curation writing – original draft, writing – review and editing. **Magdalena Annersten Gershater:** methodology, formal analysis, data curation writing – original draft, writing – review and editing. All the authors read and approved the final manuscript.

## Ethics Statement

The Swedish Ethical Review Authority approved this study 2020‐08‐03 (Reg. No. 2020‐02175), and the study followed the Helsinki Declaration of Medical Research Ethics and Standards. All participants received detailed cover letters informing them about the project and giving them the researchers' contact details.

## Consent

Written informed consent was given by all participants prior to participation.

## Conflicts of Interest

The authors declare no conflicts of interest.

## Data Availability

The data that support the findings of this study are available from the corresponding author upon reasonable request.

## Appendix 1 Open Question Concept Mapping

Concept Mappingin the study ‘Optimised care of persons with diabetes and foot complications within primary healthcare’

Your email: ………………………………………………………………………

You are asked to react to and complete an incomplete statement and turn it into 2–5 comprehensible sentences. The National Board of Health and Welfare recommends that persons with diabetes have an annual foot examination The aim is to maintain good foot health. Persons with an increased risk of developing foot ulcers should, among other things be treated with medical foot care, have appropriate shoes, and get information about self‐care. Below is an incomplete sentence for you to complete with your own words.

To improve podiatry and self‐care of the feet of in diabetes, I think that…

1………………………………………………………………………………

………………………………………………………………………………

2………………………………………………………………………………

………………………………………………………………………………

3………………………………………………………………………………

………………………………………………………………………………

4………………………………………………………………………………

………………………………………………………………………………

5………………………………………………………………………………

………………………………………………………………………………

## Appendix 2 Examples of Ideas in the Clusters

Examples of ideas in cluster 1. Examination of the feet by healthcare professionals

**Table:**

No.	Idé (Svenska)	Idea (English)
1	Tidigt skede…börja med undersökning av fötter	Start with an examination of the feet at an early stage.
11	Undersökning varje år	Annual examination
24	Vården ska ha en personcentrerad dialog om mina fötter	The healthcare provider should have a person‐centred dialogue with me about my feet.

Examples of ideas in cluster 2. Exchange of knowledge/Exchange of experiences

**Table:**

No.	Idé (Svenska)	Idea (English)
2	Vården bör informera om vikten av god fothälsa	The healthcare provider should inform about the importance of good foot health.
3	Personer över 50 år, borde få info vid läkarbesöket om förebyggande av skador på fötter (ex. skor, vård)	People over 50 years old should receive information during their doctor's visit about how to prevent foot injuries (e.g., shoes, care).
4	Få information	Get information

Examples of ideas in cluster 3. Exchange of knowledge/Exchange of experiences

**Table:**

No.	Idé (Svenska)	Idea (English)
9	Man ska få fotvård en ggr/kvartalet (t.ex. hjälp med att klippa naglar)	One should receive podiatry once a quarter (e.g., help cutting toenails)
15	Regelbundet bli kallad till vården	Regular healthcare appointments
16	Alla diabetiker ska få fotvård flera gånger om året	All patients with diabetes should receive podiatry several times a year.

Examples of ideas in cluster 4. Economy/Equal care

**Table:**

Nr.	Idé (Svenska)	Idea (English)
10	Man bör få fri tillgång till fotsalvor på recept	One should have free access to foot creams on prescription.
13	Man bör få tillgång till bättre skor, inlägg	One should have access to better shoes, insoles.
26	MyFoot kan vara ett hjälpmedel	MyFoot can be an aid.

Examples of ideas in cluster 5. Other/Support for self‐care

**Table:**

No.	Idé (Svenska)	Idea (English)
12	Bör rekommendera lämpliga inlägg, t.ex. mellan tårna.	12 HCPs should recommend suitable insoles, e.g., between the toes.
14	Man bör få tillgång till Ortoped	14 One should have access to an orthopaedist.
17	Få hjälp med utprovning av inlägg av ortopedteknisk personal	17 One should gethelp fitting insoles by personnel at the Department of Prosthetics and Orthotics.
